# Supplementary material for: Transcriptome Analysis Reveals Vimentin-Induced Disruption of Cell–Cell Associations Augments Breast Cancer Cell Migration
Source: Cells. 2022 Dec 13;11(24):4035. doi: 10.3390/cells11244035 (PMC9776984; doi:10.3390/cells11244035)
Supplement: Supplementary file 1 [file cells-11-04035-s001.zip › DEGs and enrichment analysis.zip!/DEGs and enrichment analysis/WT_VIMvsVector KEGG/GSEA/WT_VIM/MATURITY_ONSET_DIABETES_OF_THE_YOUNG/MATURITY_ONSET_DIABETES_OF_THE_YOUNG(HSA04950).html]

Details for gene set MATURITY\_ONSET\_DIABETES\_OF\_THE\_YOUNG(HSA04950)[GSEA]

|  || Dataset | fpkm.sample |
| Phenotype | sample.cls |
| Upregulated in class | WT\_VIM |
| GeneSet | MATURITY\_ONSET\_DIABETES\_OF\_THE\_YOUNG(HSA04950) |
| Enrichment Score (ES) | 0.72049844 |
| Normalized Enrichment Score (NES) | 1.188503 |
| Nominal p-value | 0.0 |
| FDR q-value | 0.23977737 |
| FWER p-Value | 0.498 |
Table: GSEA Results Summary

  

Fig 1: Enrichment plot: MATURITY\_ONSET\_DIABETES\_OF\_THE\_YOUNG(HSA04950)      
 Profile of the Running ES Score & Positions of GeneSet Members on the Rank Ordered List

  

| PROBE | DESCRIPTION (from dataset) | GENE SYMBOL | GENE\_TITLE | RANK IN GENE LIST | RANK METRIC SCORE | RUNNING ES | CORE ENRICHMENT || 1 | ENSG00000106331 | PAX4 |  |  | 178 | Infinity | -0.0025 | Yes |
| 2 | ENSG00000125820 | NKX2-2 |  |  | 225 | Infinity | -0.0029 | Yes |
| 3 | ENSG00000125798 | FOXA2 |  |  | 398 | Infinity | -0.0053 | Yes |
| 4 | ENSG00000101076 | HNF4A |  |  | 463 | Infinity | -0.0059 | Yes |
| 5 | ENSG00000121351 | IAPP |  |  | 599 | Infinity | -0.0077 | Yes |
| 6 | ENSG00000254647 | INS |  |  | 2028 | Infinity | -0.0314 | Yes |
| 7 | ENSG00000106633 | GCK |  |  | 2606 | 8.007 | 0.3333 | Yes |
| 8 | ENSG00000116833 | NR5A2 |  |  | 3118 | 4.465 | 0.5334 | Yes |
| 9 | ENSG00000170608 | FOXA3 |  |  | 5049 | 2.376 | 0.6119 | Yes |
| 10 | ENSG00000152804 | HHEX |  |  | 7495 | 1.716 | 0.6508 | Yes |
| 11 | ENSG00000135100 | HNF1A |  |  | 10469 | 1.318 | 0.6621 | Yes |
| 12 | ENSG00000182759 | MAFA |  |  | 10619 | 1.303 | 0.7205 | Yes |
| 13 | ENSG00000007372 | PAX6 |  |  | 18507 | 0.771 | 0.6231 | No |
| 14 | ENSG00000180535 | BHLHA15 |  |  | 20925 | 0.639 | 0.6121 | No |
| 15 | ENSG00000114315 | HES1 |  |  | 22995 | 0.506 | 0.6008 | No |
| 16 | ENSG00000164749 | HNF4G |  |  | 26826 | 0.142 | 0.5427 | No |
| 17 | ENSG00000163581 | SLC2A2 |  |  | 28930 | 0.000 | 0.5071 | No |
| 18 | ENSG00000139515 | PDX1 |  |  | 30683 | 0.000 | 0.4775 | No |
| 19 | ENSG00000258197 | NKX2-2-AS1 |  |  | 32064 | NaN | 0.4546 | No |
| 20 | ENSG00000185002 | RFX6 |  |  | 35622 | NaN | 0.3949 | No |
| 21 | ENSG00000283932 | AL121722.1 |  |  | 38219 | NaN | 0.3514 | No |
| 22 | ENSG00000275410 | HNF1B |  |  | 41492 | NaN | 0.2965 | No |
| 23 | ENSG00000122859 | NEUROG3 |  |  | 44835 | NaN | 0.2405 | No |
| 24 | ENSG00000163623 | NKX6-1 |  |  | 48281 | NaN | 0.1827 | No |
| 25 | ENSG00000143627 | PKLR |  |  | 51386 | NaN | 0.1306 | No |
| 26 | ENSG00000228611 | HNF4GP1 |  |  | 54587 | NaN | 0.0769 | No |
Table: GSEA details [plain text format]

  

Fig 2: MATURITY\_ONSET\_DIABETES\_OF\_THE\_YOUNG(HSA04950)      
 Blue-Pink O' Gram in the Space of the Analyzed GeneSet

  

Fig 3: MATURITY\_ONSET\_DIABETES\_OF\_THE\_YOUNG(HSA04950): Random ES distribution      
 Gene set null distribution of ES for **MATURITY\_ONSET\_DIABETES\_OF\_THE\_YOUNG(HSA04950)**
